# A simple capacitive method to evaluate ethanol fuel samples

**DOI:** 10.1038/srep43432

**Published:** 2017-02-27

**Authors:** Tatiana P. Vello, Rafael F. de Oliveira, Gustavo O. Silva, Davi H. S. de Camargo, Carlos C. B. Bufon

**Affiliations:** 1 Brazilian Nanotechnology National Laboratory (LNNano), CNPEM, 13083-970, Campinas, SP, Brazil; 2 Department of Physical Chemistry, Institute of Chemistry (IQ), UNICAMP, 13084-862, Campinas, SP, Brazil; 3 Institute of Physics “Gleb Wataghin” (IFGW), UNICAMP, 13083-859, Campinas, SP, Brazil

## Abstract

Ethanol is a biofuel used worldwide. However, the presence of excessive water either during the distillation process or by fraudulent adulteration is a major concern in the use of ethanol fuel. High water levels may cause engine malfunction, in addition to being considered illegal. Here, we describe the development of a simple, fast and accurate platform based on nanostructured sensors to evaluate ethanol samples. The device fabrication is facile, based on standard microfabrication and thin-film deposition methods. The sensor operation relies on capacitance measurements employing a parallel plate capacitor containing a conformational aluminum oxide (Al_2_O_3_) thin layer (15 nm). The sensor operates over the full range water concentration, i.e., from approximately 0% to 100% vol. of water in ethanol, with water traces being detectable down to 0.5% vol. These characteristics make the proposed device unique with respect to other platforms. Finally, the good agreement between the sensor response and analyses performed by gas chromatography of ethanol biofuel endorses the accuracy of the proposed method. Due to the full operation range, the reported sensor has the technological potential for use as a point-of-care analytical tool at gas stations or in the chemical, pharmaceutical, and beverage industries, to mention a few.

The limited amount of fossil fuels in the globe and their respective pollution concerns have led to the search for greener and efficient alternatives for energy production^1^,^2^. Among the possibilities, ethanol biofuel has been one of the main choices for combustion engines due to its suitable energy efficiency (about 70% of the gasoline power per gallon) along with lower greenhouse gas emission rates^1^,^2^. In addition, ethanol can be produced from renewable sources such as corn and sugarcane, which helps to reduce the emission of air pollutants by means of crop carbon sequestration^2^. In the United States, ethanol has been mainly used as a gasoline additive (usually at 10% vol.), while in Brazil flexible cars are able to run with either gasoline/ethanol mixture (about 25% vol. of anhydrous ethanol fuel - AEF) or simply ethanol (hydrated ethanol fuel - HEF). The main issue in ethanol as vehicle fuel, however, is its ease of adulteration by the addition of water above the standard values, which may cause engine malfunction^3^. The fraud is difficult to notice given the high water/ethanol miscibility and colorless aspect of the mixture. According to the Brazilian regulatory agency, the water content in AEF cannot exceed 0.5% vol., while the hydrated fuel must not contain more than 6.0% vol. of water^4^. In Brazil, the addition of water in ethanol fuel above the standards is considered illegal, resulting in the application of a fine and other administrative punishments. The contamination of ethanol by water is a major concern also during its distillation procedure, which is critical for fuel production as well as in chemical, pharmaceutical and beverage industries. In this scenario, the development of simple, accurate and fast methods for ethanol evaluation is of major importance.

Ethanol can be evaluated, for example, at gas stations, using a hydrometer. Although simple and fast, such method is subject to human errors during the reading of the density values. The standard laboratory method for the determination of water in ethanol is the Karl-Fischer titration (ASTM E203), which although accurate, is slow, destructive and requires skilled personnel^5^. In this sense, alternative methods for the evaluation of ethanol have been developed, such as optical sensors and spectroscopic methods^6^,^7^,^8^,^9^,^10^, mass-sensitive detectors^11^ and ultrasonic techniques^12^. Some of these, however, are complex, expensive, or cannot be made portable for point-of-care usage. Electrical/electrochemical techniques, on the other hand, have been proven to be suitable to monitor analytes in a simpler, cheaper and accurate way^3^,^13^,^14^. Among these, capacitive devices are particularly interesting, because the sensor response can be evaluated at different frequencies, depending on the analyte dielectric properties, and whether the event of interest occurs at the electrode surface or in the solution bulk^15^. Bueno and Paixão, for example, evaluated the water content in ethanol by means of capacitance measurements using an electronic tongue system^14^. The reported method, however, has shown to be restricted to water proportions of 5–25% vol., which is a very limited range of water percentage with a relatively high minimum concentration (5% vol.). In addition, their approach utilizes bare copper electrodes and ultrapure water as the contaminant. All these characteristics make the use of such devices limited for real applications. De Queiroz *et al*., on the other hand, utilized electronic tongue units coated with different materials to determine the content of tap water in ethanol, which is more realistic considering fuel adulteration, for instance^3^. From capacitance measurements, they were able to quantify the water content varying from 0% to 20% vol. in real ethanol biofuel samples.

In the following, we report the evaluation of ethanol employing a novel capacitive sensor concept based on a nanostructured solid-liquid interface. The determination of tap water in ethanol is described for the full range water concentration (approximately from 0 to 100% vol.). The reported method relies on the impedance measurements of devices containing Ni electrodes coated with a nanostructured and conformational aluminum oxide (Al_2_O_3_) film prepared by atomic layer deposition (ALD). Depending on the water/ethanol concentration, different frequencies can be used to precisely evaluate the sensor response. Such variable frequency operation provides different sensor sensitivities, which can be properly selected according to the user’s need. Here, capacitive devices have shown the capability to detect water traces in anhydrous ethanol down to 0.5%, with a limit of quantification (LoQ) of 3%. The sensor operation is simple, fast and non-destructive. Finally, we employed such sensor in the evaluation of ethanol biofuel acquired from different gas stations. The sensor response was contrasted with the results of gas chromatography (GC) analyses obtained from an accredited analytical laboratory, which confirm the accuracy of the reported method.

## Results and Discussion

**Figure 1:**
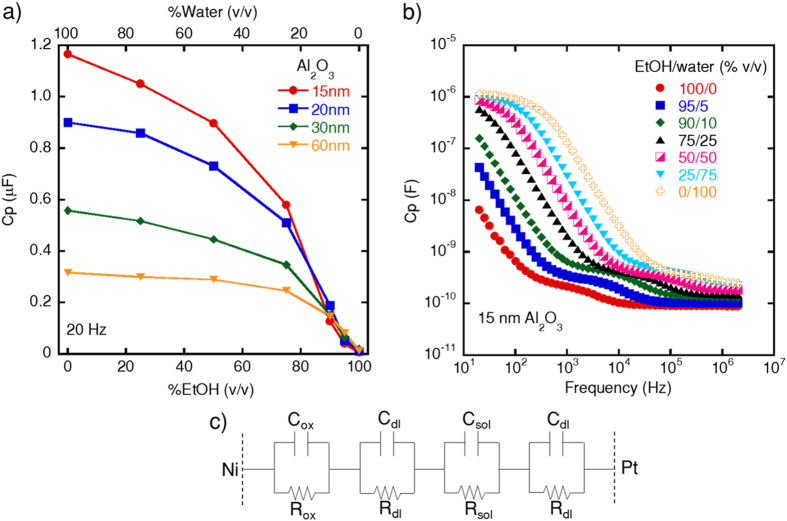
Electrical characterization of the device in ethanol. (**a**) Device capacitance (at 20 Hz) as a function of the water/EtOH concentration for different oxide thicknesses. (**b**) Capacitance as a function of frequency for different EtOH/water concentrations using a 15 nm-thick Al_2_O_3_-coated electrode. (**c**) The device equivalent circuit. The indexes ox, dl and sol correspond, respectively, to the capacitive and resistive contributions of the oxide, the electrical double layer and the solution bulk to measured impedance.

The capacitive device is composed of a parallel plate capacitor containing in one of the plates a thin film of oxide dielectric. Here, Al_2_O_3_ has been chosen as the dielectric material to coat electrodes because of its good chemical and thermal stability, large bandgap (~8.8 eV), dielectric permittivity (~9), and the possibility to obtain nanometric and conformational coatings by ALD^16^. The thickness of Al_2_O_3_ thin-films deposited by ALD has been reported as key for the sensitivity of capacitive sensors^17^. Thus, we evaluated the device response in different anhydrous ethanol (EtOH)/water mixtures varying the thickness of the Al_2_O_3_ film in the sensor active area. Solutions at different concentrations were prepared by mixing known contents of tap water and EtOH. The amount of ethanol (or water) is expressed in terms of the volume percent of total solution (water/ethanol mixture). Figure 1a exhibits the measured capacitance (C_p_) at 20 Hz as a function of the water/EtOH concentration for 15, 20, 30 and 60 nm of Al_2_O_3_. At this particular frequency and for EtOH concentrations ≥90%, all samples presented the same capacitance values. The increase of the water content leads to distinct sensor response for each Al_2_O_3_ thickness. Thin films (e.g. 15 nm) have shown to be more susceptible to variations in the water/EtOH concentration than thicker layers (viz. 60 nm) where nearly constant C_p_ values are observed for a wide range of %EtOH. According to the literature, at low frequencies (<100 Hz), charged species in the water (OH^−^, H_3_O^+^ and ionic impurities) have enough time to drift and form electrical double layers at the interface of metal electrodes in solution^18^. For electrodes coated with ultrathin oxide films, the electric double layer capacitance (C_dl_) has been reported to be non-negligible^17^. In addition, C_dl_ is known to be proportional to the solution ionic strength^19^. Thus, by increasing the content of tap water (8.6 kΩ cm) in the water/EtOH mixture, i.e., the concentration of ionic species in the sensing medium, C_dl_ is expected to increase. From Fig. 1a, such changes are observed with a more pronounced response in capacitors containing 15 nm of Al_2_O_3_. For thick Al_2_O_3_ films (e.g. 60 nm), changes in C_dl_ cannot be detected because of the oxide capacitance (C_ox_), shown in the equivalent circuit of Fig. 1c, prevails over C_dl_. In this case, C_p_ becomes approximately C_ox_ and a precise discrimination of the water content cannot be achieved. Thus, to detect small variations in the water/EtOH concentration, capacitive sensors containing ultrathin Al_2_O_3_ films are more suitable. Among the investigated thicknesses, devices containing 15 nm of Al_2_O_3_ exhibited the best response (Fig. 1a). Oxide layers thinner than 15 nm were not used to avoid possible film degradation^20^. No significant differences in C_p_ among the tested thicknesses have been observed at higher frequencies (Fig. S1). Finally, for devices absent of the nanostructured Al_2_O_3_ coating, small additions of water in highly concentrated EtOH solutions do not provide significant capacitance changes as those found using Al_2_O_3_-coated electrodes (Fig. S2). For variations from 0% to 5% vol. of water, for example, capacitance changes (∆C_p_) have been found to be 15 times greater for Al_2_O_3_-coated sensors in respect to uncoated ones at 20 Hz (Fig. S2).

As the Al_2_O_3_ conformational coating plays an import role in the evaluation of water/EtOH mixtures, we assessed the frequency-dependent response of the sensing unit employing a 15 nm-thick Al_2_O_3_. Figure 1b shows C_p_ as a function of frequency (20 Hz–2 MHz) for different water/EtOH mixtures. For samples absent of EtOH, C_p_ exhibits a plateau at low frequencies (<100 Hz), which according to the equivalent circuit in Fig. 1c can be ascribed to the series sum of C_ox_ and C_dl_. Such capacitance plateau occurs at lower frequencies (100 mHz) for the different EtOH concentrations, including 100%EtOH (Fig. S2). This suggests that C_p_ is approximately C_ox_ at very low frequencies. Thus, from the capacitance plateau (1.1 μF) we can retrieve the Al_2_O_3_ relative permittivity (ε_ox_) using Eq. 1, where ε_0_ is the vacuum permittivity, d is the oxide layer thickness (15 nm) and A the device active area (2.05 × 10^−4^ m^2^). From Eq. (1), we obtain ε_ox_ = 9.1, which is in agreement with the literature for Al_2_O_3_ films^21^,^22^. The possibility to retrieve the oxide relativity permittivity from the capacitance curves corroborates the data interpretation method.


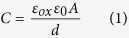


The increase of EtOH concentration, i.e., reduction in the tap water content, shifts the capacitance curves towards lower frequencies (Fig. 1b). This is considered a direct consequence of the increase in the solution resistance, as the concentration of ions in the medium reduces (Fig. S3). Such evaluation can be further confirmed by measuring different water/EtOH mixtures using resistive (18 MΩ cm) deionized water (Fig. S4). From Fig. 1b, capacitance changes are particularly evident in the 20 Hz–2 kHz frequency range. At high frequencies (10^6^ Hz), ions in solution are not able to follow the electric field oscillations and C_p_ becomes the device geometric capacitance with the water/EtOH solution acting as the dielectric medium (C_sol_). From Fig. 1b, the sum of C_ox_ and C_sol_ in a series association is approximately C_sol_, given the respective thicknesses (15 nm for the Al_2_O_3_ layer and 620 μm for the plate interspace) and relative dielectric permittivities. Therefore, from the capacitance curves at 10^6^ Hz for both 100% EtOH and 100% water samples, we can retrieve the relative permittivity of EtOH (ε_EtOH_) and water (ε_H2O_), respectively. Replacing in Eq. (1) ε_ox_ by the relative permittivity of the respective solutions, and d by the plate spacing (620 μm), we find ε_EtOH_ = 29 and εH2O = 76. These values are in reasonable agreement with the literature, where ε_EtOH_ = 25 and ε_H2O_ = 79 are found^23^.

**Figure 2:**
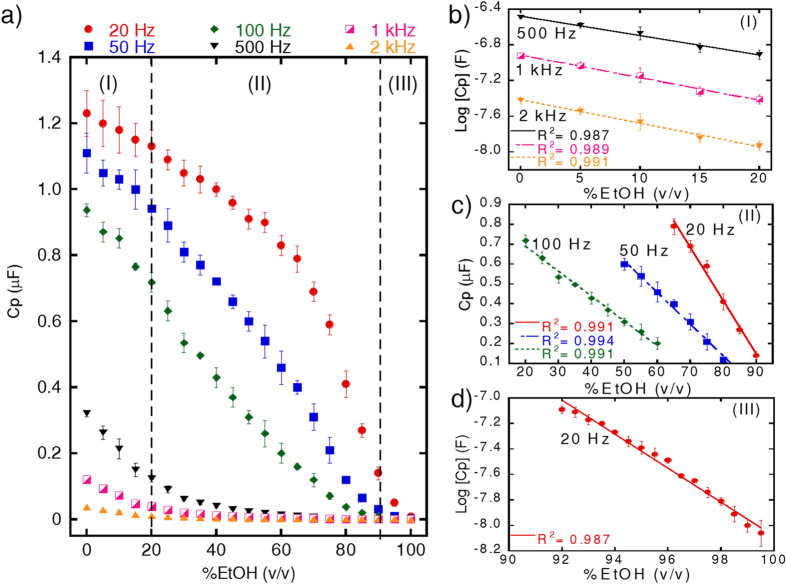
Sensor response for different ethanol/water mixtures. (**a**) Cp as a function of the EtOH content (from 0% to 100% vol.). The device response can be sectored in three regions: region I for %EtOH from 0% to 20% vol., region II from 20% to 90% vol. and region III for EtOH concentrations higher than 90% vol. Calibration curves for the device response in the respective regions (**b**–**d**). The error bars correspond to the standard deviation values obtained using six similar devices.

After the device electrical characterization, we evaluated the sensor response in EtOH/water mixtures at different frequencies, as shown in Fig. 2. Six frequency points (20 Hz, 50 Hz, 100 Hz, 500 Hz, 1 kHz and 2 kHz) have been considered to determine the best device operation range. Here, we observe that the device behaves differently at each frequency depending on the water/EtOH concentration. From Fig. 2a, we notice the device response sectored in three operation regions according to the water/EtOH concentration. Region I accounts for EtOH concentrations ranging from 0% to 20% vol.; region II is related to %EtOH varying from 20% to 90% vol.; and region III accounts for %EtOH higher than approximately 90% vol. From the device’s response, we can obtain calibration curves for each of the above-mentioned regions, as shown in Fig. 2b–d. The error bars in the curves correspond to the standard deviation values obtained using six similar devices.

From Fig. 2b, we observe that three distinct frequencies, namely 500 Hz, 1 kHz and 2 kHz, provide a similar sensor response for the %EtOH varying from 0% to 20% vol. The three curves present approximately the same slope, i.e., the same sensor sensitivity, which is defined by the amplitude of the output signal per percent of EtOH. From Fig. 2b, we find a mean sensitivity of −0.02 F/%EtOH (in the semi-log scale) for the analyzed frequencies. For %EtOH varying from 20% to 90% vol., a linear response (R^2^ > 0.99) can be obtained in 20 Hz, 50 Hz, and 100 Hz, depending on the EtOH concentration range (Fig. 2c). At such frequencies, the sensor sensitivity is −2.8 × 10^−8^ F/%EtOH (20 Hz), −1.7 × 10^−8^ F/%EtOH (50 Hz) and −1.2 × 10^−8^ F/%EtOH (100 Hz). Each calibration curve was determined in frequencies where the calculated sensitivity is the highest found for the respective EtOH concentration range. Such variable frequency operation allows one to choose the desired frequency according to the EtOH/water composition in the envisioned application. For an unknown water content, a first screening at the high-frequency region is suggested, followed by the water determination using one of the calibration curves shown. For %EtOH > 90% vol. (Fig. 2d), which is of interest in the evaluation of fuel samples, a sensitivity of −0.13 F/%EtOH (in the semi-log scale) is obtained. Given the sensor sensitivity and the capability to easily to select the operation region, the developed platform may be used as a universal tool to monitor EtOH/water mixtures. From Fig. 2d, we determined the sensor limit of detection (LoD) for water in EtOH as being 0.5% and the LoQ as 3%. In the literature, just a few sensors present similar characteristics that combine operation over full range concentration with low detectable amounts of water. Such devices include electrical sensors based on ZnO nanorod arrays^13^ and optical fiber-based sensors^7^,^8^,^9^,^10^. Our device, however, is simpler than the reported methods, in addition to being responsive to tap water, which is more realistic considering fuel adulteration. Finally, we evaluated the sensor response in the presence of methanol to emulate real fuel samples. According to the regulation^4^, the maximum amount of methanol allowed in bioethanol fuel is 0.5% vol. Thus, we have carried out measurements in highly concentrated ethanol solutions (94.5–95%) having 0.5% vol. of methanol and 4.5–5.0% vol. of water. No differences in the sensor response have been found among samples where 0.5% vol. of methanol is present or absent (Fig. S5). This indicates that methanol, at low concentrations, is not an interferent in the operation of the reported sensor. The proposed method, therefore, possesses the capabilities necessary to evaluate HEF samples, as discussed in the following.

The capacitive sensor was employed in the evaluation of real ethanol biofuel samples acquired from different gas stations. Three identical devices were employed in the analysis of HEF. The sensor response was compared with the results obtained from GC (two measurements). Finally, two HEF samples were also intentionally adulterated with water and further analysed by GC. The results are expressed in terms of the ethanol content, as shown in Table 1.

From Table 1, the proposed platform has shown to be very precise, presenting low standard deviation values (in average 0.5%). The capacitive devices are also accurate, exhibiting deviations from 1% to 2.7% with respect to the results obtained by GC. Such discrepancies can be attributed to the presence of adventitious contaminants in the fuel (e.g., additives) rather than simply water, in which the reported sensor is able to detect at concentrations down to 0.5% vol. The method described here, however, is simpler and cheaper than GC. In contrast to GC, it does not need skilled personnel for the evaluation of samples, it is a non-destructive technique and it can be made portable for point-of-care analysis of fuel, for example, at gas stations. Regarding the literature for the evaluation of real fuel samples, our sensor is more accurate than the device reported by Bueno and Paixão^14^. In addition, their lowest quantifiable amount of water is 10% vol., which is higher than the minimum concentration allowed in HEF according to the Brazilian regulation (viz. 6% vol.)^4^. Our device, on the other hand, is able to detect water concentrations below such a value. De Queiroz *et al*.^3^ have also shown to be able to detect such low amounts of water; however, no details about their sensor accuracy have been reported.

The time required for the evaluation of ethanol samples is short, varying from 2 to 15 min. The first analysis requires the sensor to remain immersed in solution for stabilization prior to the electrical measurements, i.e. for a complete frequency sweep (20 to 2 MHz). The whole procedure takes approximately 15 min. Further analyses are faster, taking 2 min to perform the measurements. The sensor has shown to be robust, enduring at least ca. 50 consecutive measurements with the same precision and accuracy reported in Table 1. In addition, the devices have lasted for a minimum of 5 months, with no special care during storage. In the case of eventual failure, the Al_2_O_3_ conformational layer can be easily regenerated without compromising neither the substrate nor the metallic parts. Since the whole device fabrication is based on standard microfabrication techniques and thin-film deposition, the sensors can be produced in large scale at low cost. Finally, we believe the reported sensor has the technological potential for the evaluation of ethanol biofuel and other applications utilizing ethanol in the chemical, pharmaceutical and beverage industries.

## Conclusion

In this work, we demonstrated a novel platform to evaluate ethanol samples based on nanostructured capacitive devices. The sensor fabrication is simple, relying solely on photolithographic and thin film deposition processes. The sensor active region is composed of a conformational Al_2_O_3_ film deposited by ALD. We evaluated the effect of different Al_2_O_3_ thickness on the detection of the water content in anhydrous ethanol samples. The evaluation of ethanol is carried out by impedance spectroscopy measurements and the results expressed in terms of the capacitance values. Sensing units containing a 15 nm-thick Al_2_O_3_ film allows the monitoring of water content in ethanol over its full range of concentration, i.e., from 0% to 100% vol. of water. To the best of our knowledge, this is the widest range of operation for the water content in ethanol reported using capacitive devices. The proposed sensor exhibited a limit of detection of 0.5% vol. of water in anhydrous ethanol, with a limit of quantification of 3%. In the evaluation of ethanol fuel, the reported device shows an excellent agreement with the results obtained from gas chromatography analyses. The method described here, however, is simpler and cheaper than gas chromatography for the analysis of ethanol. In addition, the reported platform can be incorporated in portable point-of-care systems, for example, to evaluate ethanol fuel along the whole supply chain. We envisage that by combining such platform with proper electronics, the very same sensor could be used to monitor the fuel quality from the biorefinery to the final customer, i.e., from its production to transportation, storage, and commercialization at gas stations. Such a possibility would allow the continuous control of the fuel integrity by real-time data reading and uploading to cloud severs. Other possibilities include the monitoring of ethanol quality, for example, in the chemical, pharmaceutical and beverage industries.

## Materials and Methods

**Figure 3:**
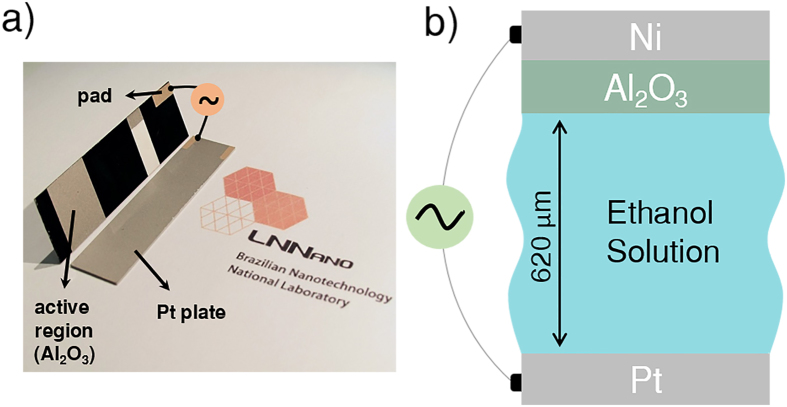
Device layout and equivalent circuit model. (**a**) Picture of the sensing device showing the two capacitor plates, (**b**) the sketch of the capacitor configuration for the evaluation of ethanol samples (not to scale).

The capacitive sensors (Fig. 3a) were fabricated by photolithography and thin-film deposition on clean alumina substrates (5.5 cm × 1.3 cm). The cleaning procedure starts with rinsing the substrates sequentially in acetone p.a., acetone VLSI (very-large-scale-integration) and in isopropanol VLSI, for 20 min each in ultrasonic bath. Following, the substrates were cleaned in piranha solution 2:1 v/v (H_2_SO_4_:H_2_O_2_) and oxygen plasma for 3 min (90 W, pressure 0.4 mbar). In one of the plates, thin films of 50 nm Cr (2 Å/s) and 200 nm Ni (1 Å/s) were deposited by e-beam evaporation using a shadow mask to pattern the metallic parts. Contact pads consisting of Cr (20 nm, 2 Å/s) and Au (50 nm, 1 Å/s) were thermally deposited in the region indicated in Fig. 3a. A 25 μm-thick film of AZ nLof 2070 photoresist was spun cast on the surface, followed by a hot plate baking (100 °C, 90 s) and UV exposure (480 mJ/cm^2^ dose) to define the sensor active area. In this region, Al_2_O_3_ films at different thicknesses (15, 20, 30 and 60 nm) were deposited by ALD at 150 °C. Al_2_O_3_ films were produced by pulsing alternately the precursor trimethylaluminum (TMA) 97%, acquired from Sigma-Aldrich, and water in the reaction chamber (Cambridge Nanotech Savannah 100 ALD System). The TMA and water reservoirs were kept at room temperature, while all other heating stages, including the sample stage, were kept at 150 °C. The pulse duration was 20 ms, with a waiting time of 15 s. The chamber pressure was set between 10^−1^–10^−2^ Torr and Argon was used as the carrier/purging gas at a flow of 5 sccm. Such fabrication conditions provided a film coverage of 1.1 Å/cycle of operation. This method ensures a conformational coating of the Ni electrode. As a result, the ethanol solution will not be in direct contact with the capacitor metallic parts, preserving its integrity. To complete the capacitor architecture, we coupled this unit to a 200 nm-thick Pt electrode in a parallel plate configuration, with a plate separation of approximately 620 μm. The fabrication steps are shown in detail in the Supplementary Information (Fig. S6). Finally, the capacitor is immersed in solution, as shown in Fig. 3b, for the evaluation of samples.

The evaluation of ethanol was carried out by electrical impedance measurements using an Agilent E4980A LCR meter. We recorded the device parallel capacitance and resistance values in the 20 Hz–2 MHz frequency range using a sine-wave voltage signal amplitude of 10 mV. All electrical measurements were performed in laboratory ambient with controlled temperature (20 ± 2 °C) and humidity (50%). The device response was analyzed in different frequencies varying the amount of tap water, from 0% to 100% vol., in anhydrous ethanol absolute (EtOH), purity ≥99.9%, acquired from Merck (Germany). The device response was modeled using the equivalent circuit shown in Fig. 1c.

The proposed platform was used to evaluate ethanol biofuel (viz. HEF) acquired from different gas stations. The sensor response was contrasted with the results of GC analyses obtained from an accredited ISO17025 analytical service laboratory. We also performed blind tests, where HEF samples were intentionally adulterated with water by a third party and the ethanol content confirmed by using the proposed device.

## Additional Information

**How to cite this article:** Vello, T. P. *et al*. A simple capacitive method to evaluate ethanol fuel samples. *Sci. Rep.* **7**, 43432; doi: 10.1038/srep43432 (2017).

**Publisher's note:** Springer Nature remains neutral with regard to jurisdictional claims in published maps and institutional affiliations.

## Supplementary Material

Supplementary Information

## Figures and Tables

**Table 1:** Comparison of responses between the proposed device and GC analyses.

Sample	Ethanol content (% vol.)		Deviation
	Sensor response	GC response	
HEF#1	94.6 (±0.5)	95.6 (±0.1)	1%
HEF#2	94.1 (±0.4)	96.4 (±0.1)	2.4%
HEF#3	94.4 (±0.5)	96.1 (±0.1)	1.8%
adulterated HEF#1	93.1 (±0.3)	94.1 (±0.1)	1.1%
adulterated HEF#2	83.5 (±0.8)	85.8 (±0.1)	2.7%
