# Supplementary material for: A simple capacitive method to evaluate ethanol fuel samples
Source: Sci Rep. 2017 Feb 27;7:43432. doi: 10.1038/srep43432 (PMC5327469; doi:10.1038/srep43432)
Supplement: Supplementary Information [file srep43432-s1.doc]

A simple capacitive method to evaluate ethanol fuel samples

Tatiana P. Vello^a,b^, Rafael F. de Oliveira^a^, Gustavo O. Silva^a^, Davi H. S. de Camargo^a^ and Carlos C. B. Bufon^a,b,c,*^

^a^ Brazilian Nanotechnology National Laboratory (LNNano), CNPEM, 13083-970, Campinas, SP, Brazil

^b^ Department of Physical Chemistry, Institute of Chemistry (IQ), UNICAMP, 13084-862 Campinas, SP, Brazil

^c^ Institute of Physics “Gleb Wataghin” (IFGW), UNICAMP, 13083-859, Campinas, SP, Brazil

*Corresponding author: cesar.bof@lnnano.cnpem.br

Supplementary Information


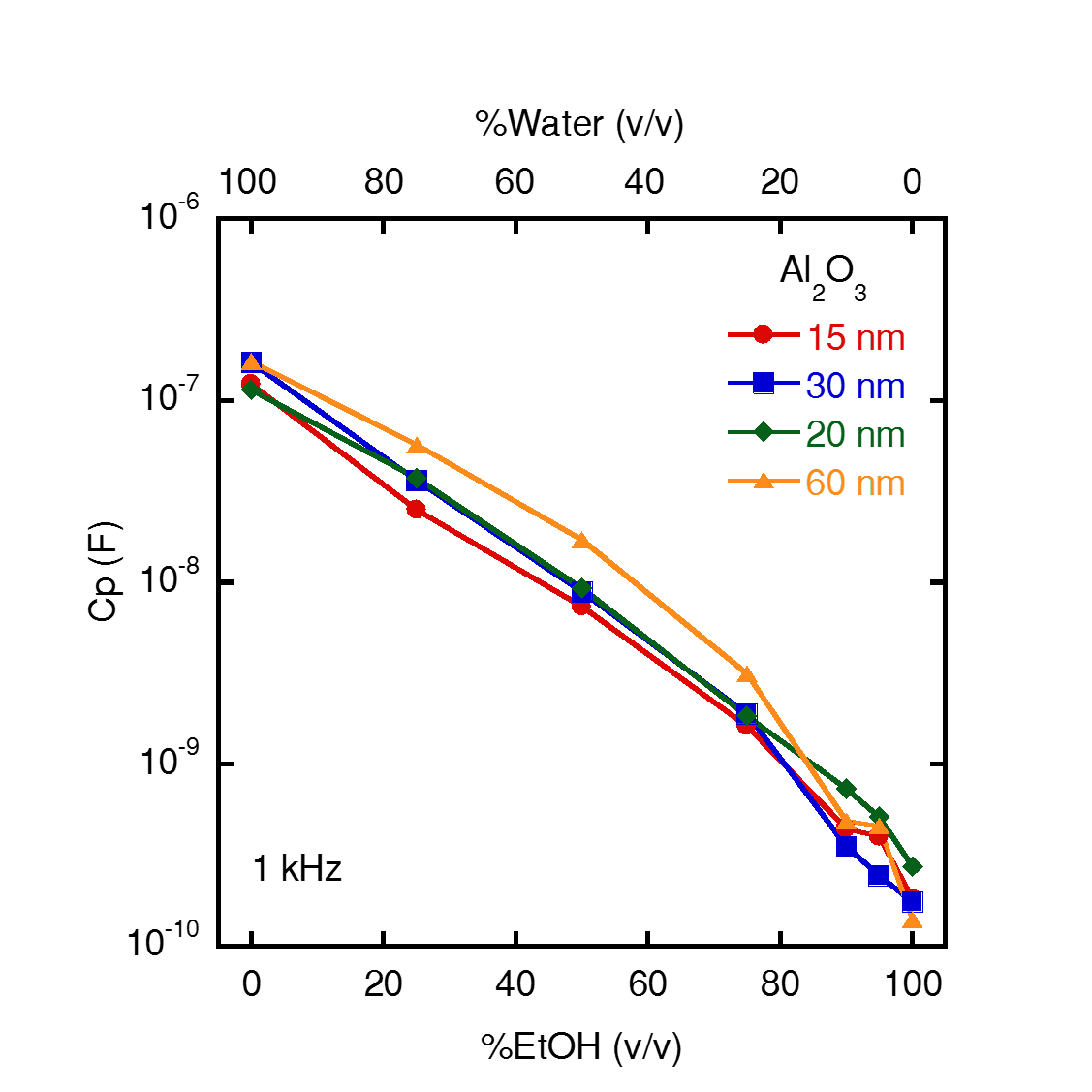


**Figure S1:** Capacitance as a function of the ethanol concentration (%EtOH) at 1 kHz for different Al_2_O_3_ thicknesses.


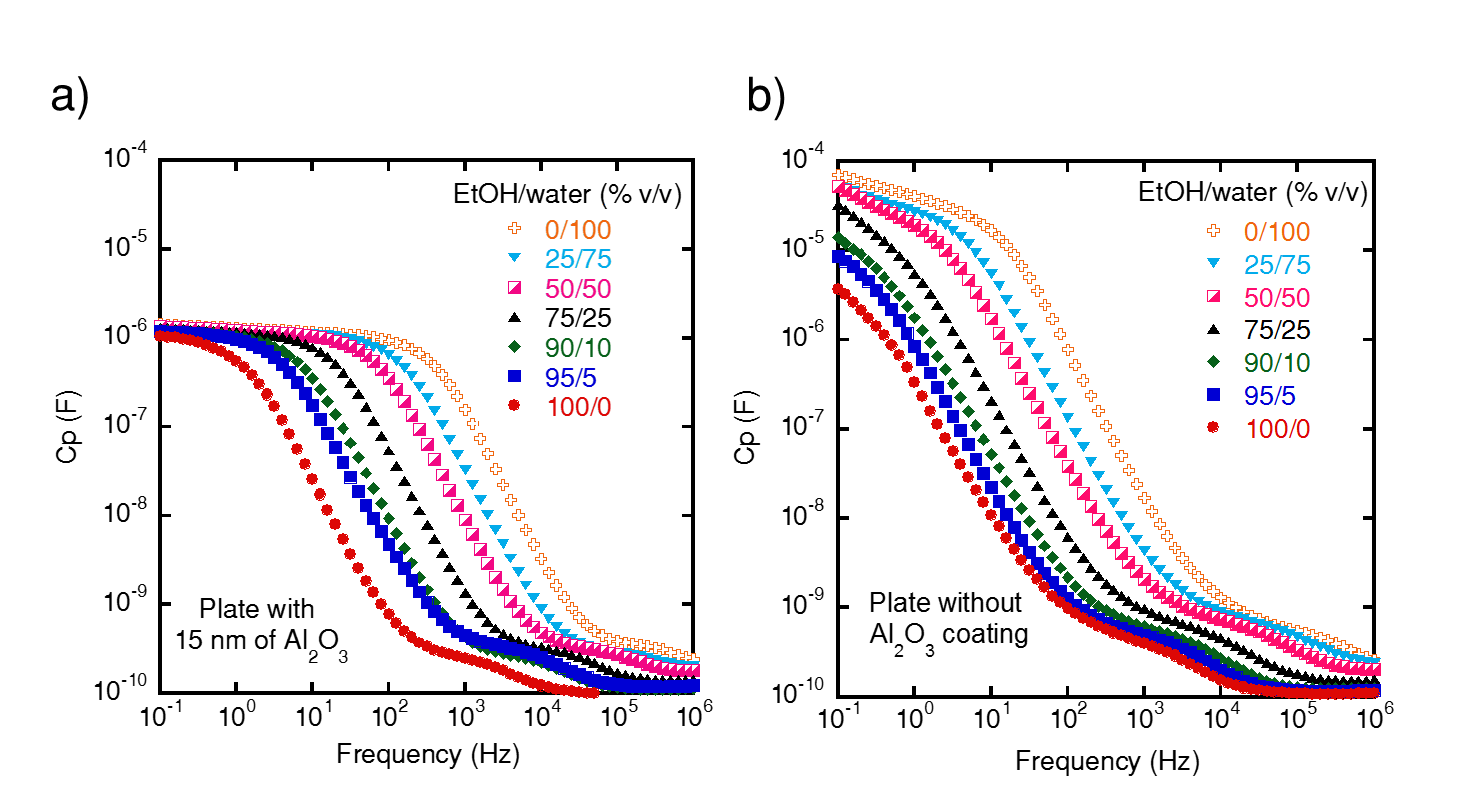


**Figure S2:** Capacitance as a function of frequency for different EtOH/water mixtures for a) coated and b) uncoated electrodes. The measurements were performed using Solartron frequency response analyzer (model 1260) coupled to a dielectric interface (model 1296) to reach very low frequencies (10^-1^ to 1 MHz). The capacitance variation is up to 15 times higher for coated units than for uncoated ones, considering low water contents (from 0% to 5% vol. of water) for the complete sensor operational frequency (20 Hz – 2 kHz).


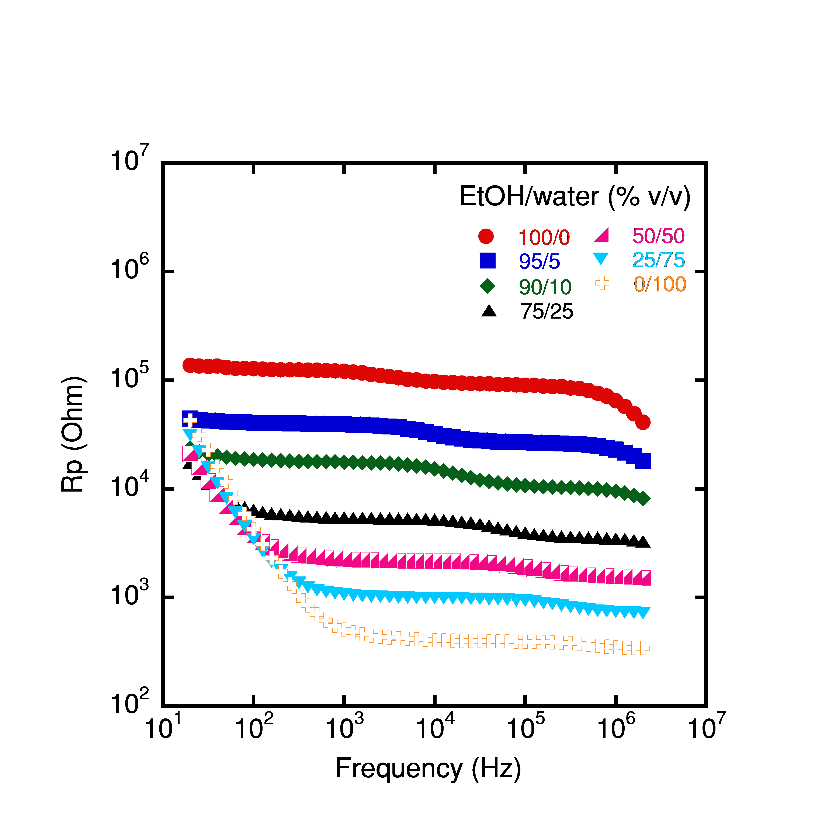


**Figure S3:** Parallel resistance (Rp) as a function of frequency for different EtOH/water mixtures using 15 nm-thick Al_2_O_3_ coated electrodes.

**
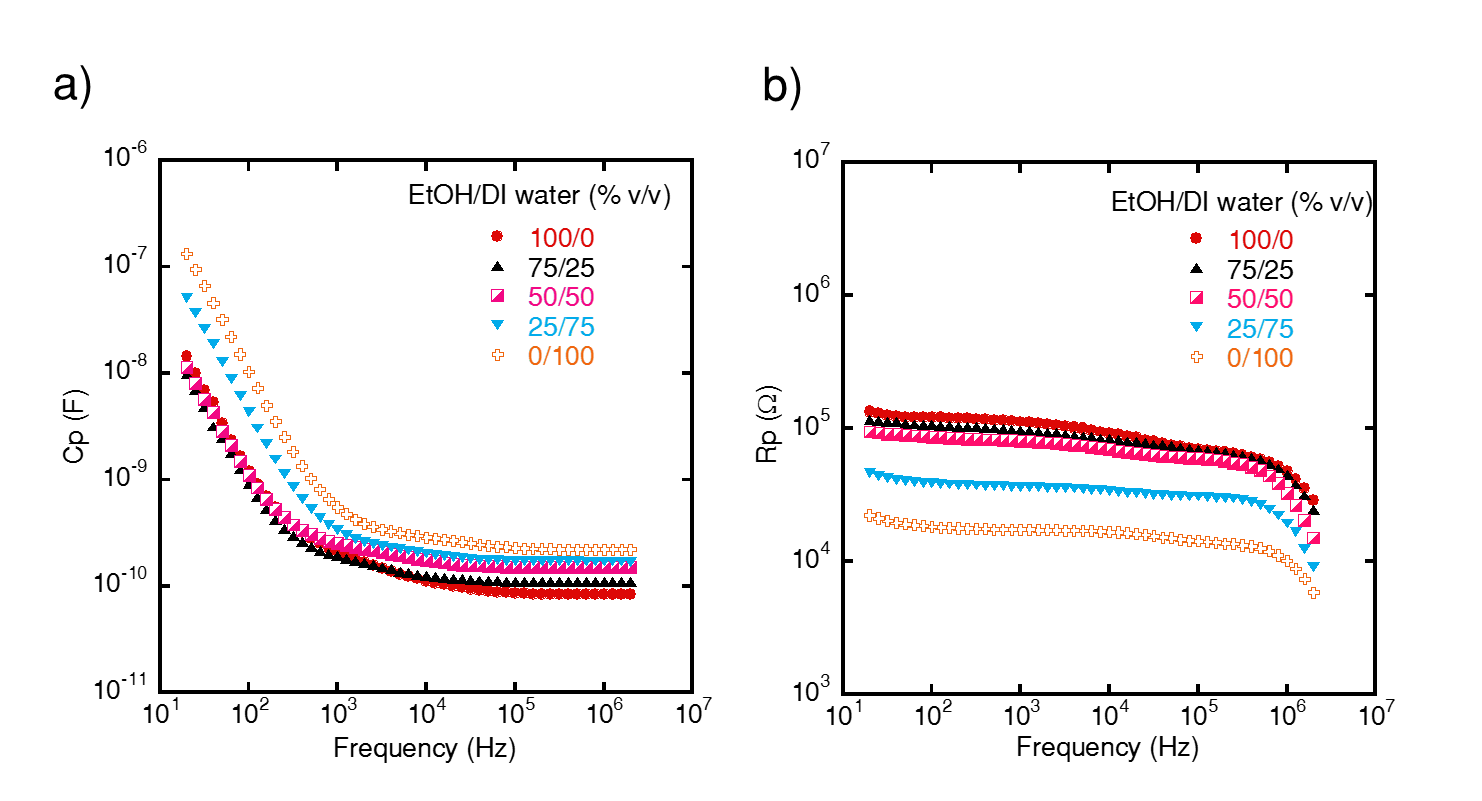
**

**Figure S4:** a) Cp and b) Rp as a function of frequency for different concentrations of EtOH/deionized water (DI) using 15 nm-thick Al_2_O_3_ coated electrodes.


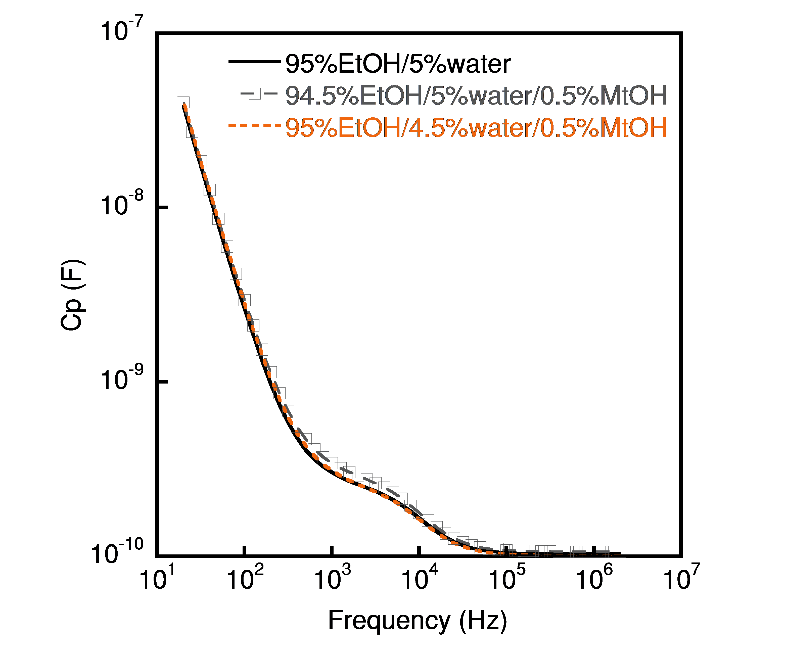


**Figure S5:** Evaluation of the sensor response towards the presence of 0.5% vol. of methanol (MtOH) as a typical contaminant of biofuels.


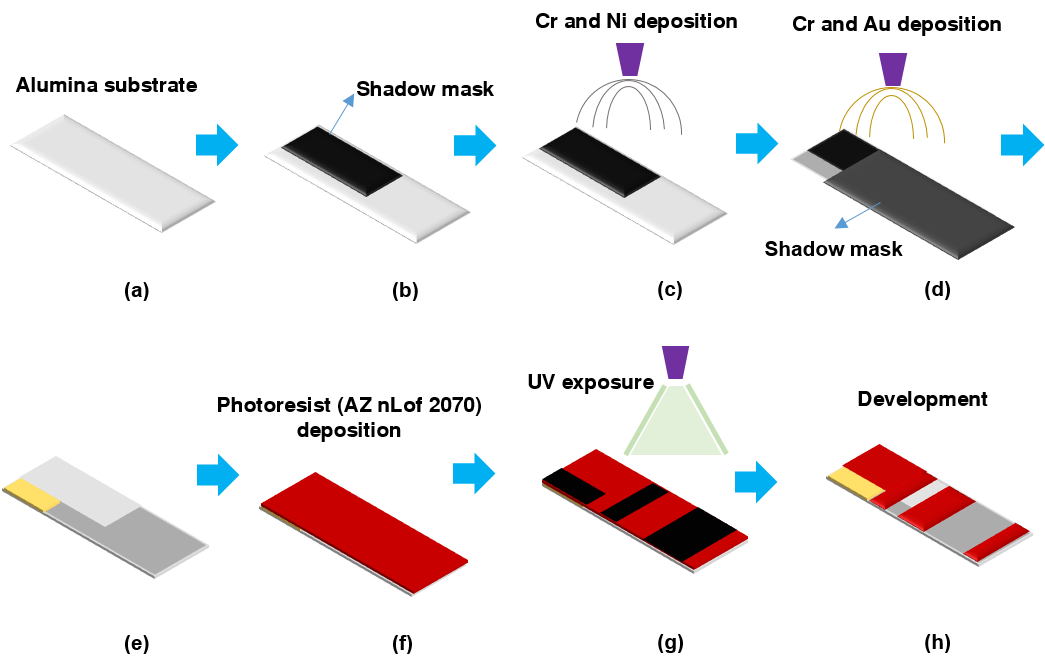


**Figure S6:** Schematic diagram of the fabrication process. a) Alumina substrate used to prepare the capacitor plate, b) electrode definition using a shadow mask, c) deposition of the Cr/Ni electrode, d) Cr and Au deposition of the contact pads, e) plate layout after electrode patterning, f) spincoating of the photoresist layer, g) photoresist exposure to UV light and h) development. The nanostructured Al_2_O_3_ film is deposited by ALD after the step (h).
